# Altered Microstructural Changes Detected by Diffusion Kurtosis Imaging in Patients With Cognitive Impairment After Acute Cerebral Infarction

**DOI:** 10.3389/fneur.2022.802357

**Published:** 2022-02-28

**Authors:** Liting Fan, Fatima Elzahra E. M. Ibrahim, Xiaoqi Chu, Yu Fu, Hongting Yan, Zheng Wu, Chunmei Tao, Xuejing Chen, Yue Ma, Yunchu Guo, Yang Dong, Chao Yang, Yusong Ge

**Affiliations:** Second Affiliated Hospital of Dalian Medical University, Dalian, China

**Keywords:** vascular cognitive impairment, acute cerebral infarction, diffusion kurtosis imaging, splenium of corpus callosum, microstructural change

## Abstract

**Objective:**

To detect the microstructural changes in patients with cognitive impairment after acute cerebral infarction using diffusion kurtosis imaging (DKI).

**Materials and Methods:**

A total of 70 patients with acute cerebral infarction were divided into two groups: 35 patients with cognitive impairment (VCI group), and 35 patients without cognitive impairment (N-VCI group), according to mini-mental state examination (MMSE) score. Healthy individuals (*n* = 36) were selected as the normal control (NORM) group. DKI parameters from 28 different brain regions of interest (ROIs) were selected, measured, and compared.

**Results:**

VCI group patients had significantly higher mean diffusion (MD) and significantly lower mean kurtosis (MK) values in most ROIs than those in the N-VCI and NORM groups. DKI parameters in some ROIs correlated significantly with MMSE score. The splenium of corpus callosum MD was most correlated with MMSE score, the correlation coefficient was −0.652, and this parameter had good ability to distinguish patients with VCI from healthy controls; at the optimal cut-off MD value (0.9915), sensitivity was 91.4%, specificity 100%, and the area under the curve value 0.964.

**Conclusions:**

Pathological changes in some brain regions may underlie cognitive impairment after acute cerebral infarction, especially the splenium of corpus callosum. These preliminary results suggest that, in patients with VCI, DKI may be useful for assessing microstructural tissue damage.

## Introduction

The concept of vascular cognitive impairment (VCI) was proposed by Hachinski et al. in 1993. VCI is a cognitive impairment syndrome caused by cerebrovascular disease and risk factors for cerebrovascular disease, which manifests as single or multiple functional impairments; for example, in language, memory, calculation, and executive ability, and the symptoms generally worsen over time (1). The incidence of cognitive decline after stroke is much higher than the stroke recurrence rate, with as many as 30% of stroke patients showing varying degrees of cognitive decline (2). VCI is currently considered the second leading cause of dementia after Alzheimer's disease (AD) (3), and has a negative impact on patient quality of life, as well as representing a considerable burden to families and society. Therefore, it is particularly important to identify VCI occurrence at an early stage, and specific examination methods for early detection of VCI are currently lacking.

Diffusion tensor imaging (DTI) was initially assumed to function based on the principle of water molecule diffusion by Gaussian motion; however, experiments have demonstrated that, in biological tissues, complex cell components and structures hinder and limit water molecule diffusion; the imaging gradient strength, direction, and temporal profile affect sensitivity to diffusion and are commonly reduced to a single simplified parameter referred to as the b-value [unit: s/mm^2^]. Therefore, at high b values, this assumption is not applicable in brain tissue (4). Diffusion kurtosis imaging (DKI) is a recently developed method for measuring non-Gaussian diffusion of water molecules. DKI parameters include both diffusion parameters [fractional anisotropy (FA), mean diffusion (MD), axial diffusion (Da), and radial diffusion (Dr)] and kurtosis parameters [fractional anisotropy kurtosis (FAK), mean kurtosis (MK), axial kurtosis (Ka) and radial kurtosis (Kr)] (5). To obtain diffusional kurtosis maps, the logarithm of the signal decay following a diffusion-sensitized sequence is described, as by the following equation (6): ln[S(b)] = ln[S(0)]-bD+1/6(b^2^D^2^K)+0(b^3^), where S(b) is the signal intensity, D is the diffusion coefficient and K is the diffusional kurtosis. When K = 0, Gaussian formalism is recovered. This equation represents a cumulant expansion for the diffusion nuclear magnetic resonance signal.

DKI can reflect the high heterogeneity in the limited diffusion of water particles in a brain tissue injury area; hence, compared with diffusion-weighted imaging (DWI) and DTI, it better reflects the heterogeneity and complexity of tissues, and is more conducive to detecting subtle changes in tissue structure under pathological conditions (7). DKI parameters are very sensitive and can identify characteristic changes in many neurological diseases (8, 9). Further, changes in DKI parameters can be detected even before they are visible using conventional imaging, and the performance of DKI is better than that of conventional DTI (10, 11). Therefore, compared with the traditional DTI imaging, DKI has a wider scope for application to detect microstructural changes (9). To date, there have been few studies on the application of DKI in nervous system diseases, and relevant studies using DKI to investigate VCI are very scarce.

Here, we aimed to detect microstructural changes in patients with cognitive impairment after acute cerebral infarction using DKI, and to further evaluate the relationship between these parameters and Mini-Mental State Examination (MMSE) score, to provide neuroimaging evidence to assist in diagnosis of patients with VCI.

## Materials and Methods

### Study Population

**Figure 1:**
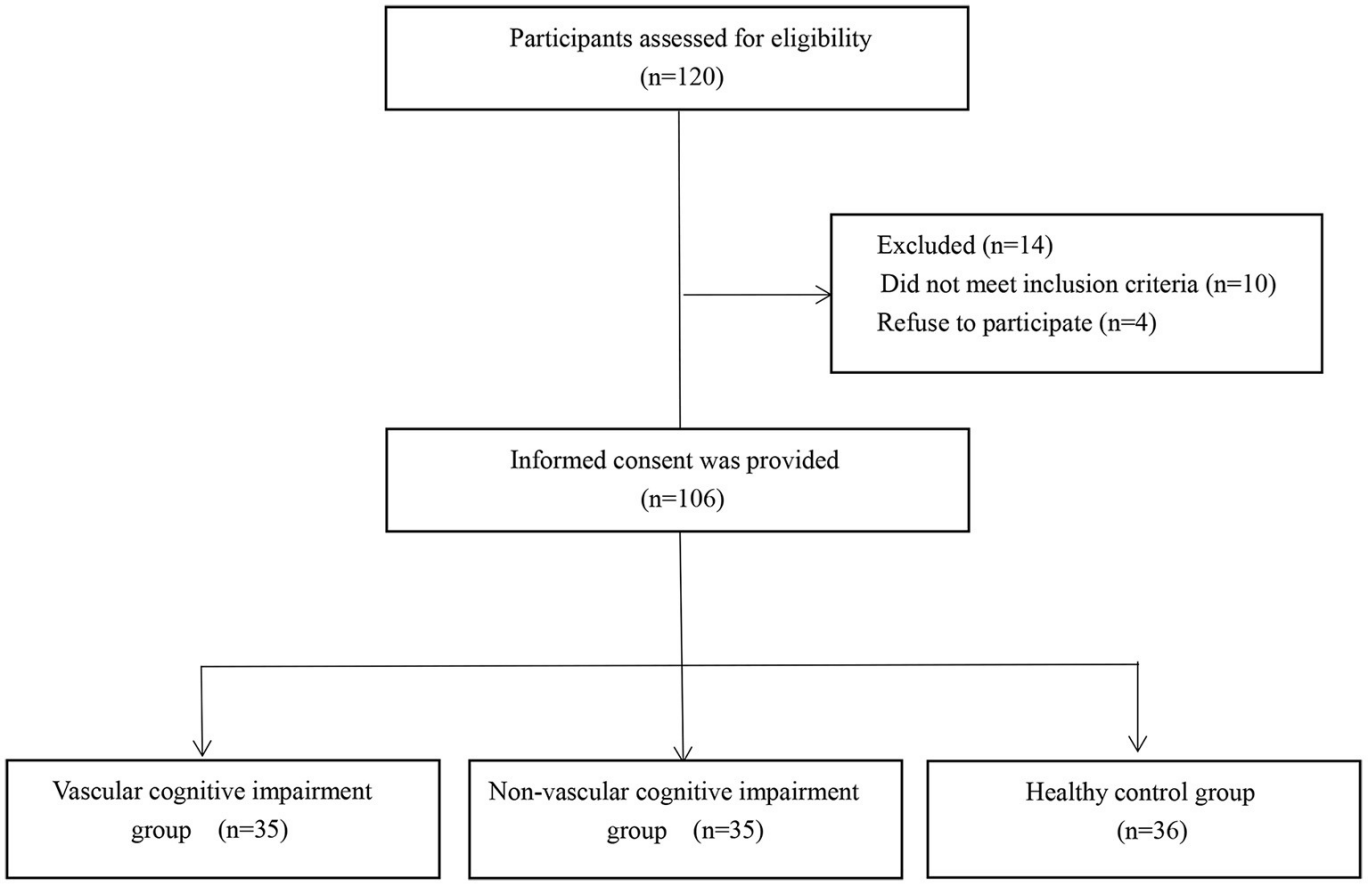
Design and flow of participants through the study.

A total of 70 patients with acute cerebral infarction were enrolled in this study in a tertiary hospital, including 35 patients with cognitive impairment (VCI group, the mean age was 66.3 years, 17 males and 18 females), and 35 patients without cognitive impairment (N-VCI group, the mean age was 63.1 years, 15 males and 20 females). The Ethics Review Board of our institution reviewed and approved this research procedure, in compliance with the Declaration of Helsinki, and informed consent was obtained from each patient. The inclusion criteria were as follows: acute onset within 7 days of cerebral infarction; symptoms of local neurological deficits; symptoms or signs lasting >24 h or any duration, but imaging showed ischemic lesions on the responsible side; and no cerebral hemorrhage according to CT or MRI examinations. The exclusion criteria were as follows: patients with dementia or severe cognitive impairment before the onset of stroke; patients with consciousness disorder, aphasia or dysarthria, or hearing impairment and unable to cooperate with the examination; severe terminal stage disease or severe disease complicated by dysfunction of vital organs; previous history of schizophrenia, severe anxiety and depression, or other mental health conditions; patients with metal materials or other implants in the body that prohibit use of magnetic resonance imaging; or refused to sign informed consent. Simultaneously, 36 healthy individuals (control group, the mean age was 61.1 years, 21 males and 15 females) with similar age were selected. The following exclusion criteria for normal controls were applied: with history of clinical stroke; severe terminal stage disease or severe disease complicated by dysfunction of vital organs; previous history of schizophrenia, severe anxiety and depression, or other mental health conditions; with obvious structural abnormalities on MRI scans; test scores of cognitive assessment were not within normal range; patients with metal materials or other implants in the body that prohibit use of magnetic resonance imaging; or refused to sign informed consent (Figure 1).

General data and stroke characteristics of the subjects were obtained on admission. The Modified Rankin Scale (MRS) and the National Institutes of Health Stroke Scale (NIHSS) were applied to assess stroke severity. Infarction areas were grouped according to the Adams classification method (12), as follows: < 1.5 cm^2^, lacunar infarction group; 1.5–3.0 cm^2^, with occlusion of small vessels involving one anatomical site, small infarction group; > 3.0 cm^2^, with occlusion of large brain arteries involving > 2 anatomical sites, large infarction group. Cerebral infarction lobes and locations were also analyzed in groups. Baseline data on age, sex, education level, hypertension, diabetes, smoking, and alcohol consumption history were collected from all subjects.

All subjects underwent venous blood sampling from the middle elbow on an empty stomach in the morning, for routine blood biochemistry, blood clotting, hypersensitive C-reactive protein, hepatic function, renal function, myocardial enzyme, fasting glucose, glycosylated hemoglobin, low-density lipoprotein cholesterol, high-density lipoprotein cholesterol, total cholesterol, triglyceride, vitamin B12, folic acid, and thyroid function test parameter investigation.

### Study Protocol

All subjects were assessed by two professionally trained neuropsychological evaluators using a neuropsychological scale during the visit. MMSE scores were used to evaluate overall cognitive function, where an MMSE score < 27 was considered objective cognitive impairment (13). MMSE scores for orientation, registration, attention, and calculation, as well as recall, language and praxis, were recorded. The Hamilton Anxiety (HAMA) and Hamilton Depression (HAMD) scales were used to assess the severity of anxiety and depression disorders.

All subjects underwent DKI scans within 4–7 days of admission. T1-weighted images, T2-weighted images, FLAIR sequences, DWI sequences, and DKI sequences were all scanned using a GE MR750W 3.0T magnetic resonance scanner. The following axial scanning parameters were used for DKI scans: repetition time, 5,000 ms; echo time, 90 ms; field-of-view, 24 × 24 cm; imaging matrix, 256 × 256; number of slices, 24; slice thickness, 5 mm; slice gap, 0 mm; b values, 0, 1,000, and 2,000 s/mm^2^; gradient directions, 15; and scan time, 5′55″. Data were analyzed using a function tool in GE AW 4.7 post-processing workstation post-processing software. Two radiologists (YD and CY, with 10 years and 17 years of experience in neuroimaging, respectively), assessed the images, drew ROIs and checked them. When two radiologists disagreed, they discussed with each other to reach a consensus. ROIs were as follows: bilateral frontal lobe, bilateral parietal lobe, bilateral temporal lobe, bilateral occipital lobe, genu of corpus callosum, splenium of corpus callosum, anterior limb of bilateral internal capsule, posterior limb of bilateral internal capsule, bilateral head of caudate nucleus, bilateral putamen, bilateral thalamus, bilateral medial temporal lobe, bilateral corona radiata, bilateral subcortical white matter, and bilateral cerebral cortex. ROI areas ranged from 10 to 30 mm^2^, according to the size of the measured anatomical structure. Mean values of FA, MD, Da, Dr, FAK, MK, Ka, and Kr in these segmented ROIs were recorded. The infarct zone, brain gray matter, cerebral sulcus, and vascular space were avoided while selecting ROIs, and attempts were made to ensure that all patients were imaged at the same level, bilateral symmetry, and area. The radiology technicians who collected MRI images were blinded from the distribution of research subject groups to avoid the error caused by subjective reasons in the process of operation and affect the final result.

### Sample Size Calculations

SPSS11.0 was used to estimate the sample size, α = 0.05, β = 0.1, bilateral test, balanced group design was adopted, and the sample size was estimated based on the MD value of the splenium of corpus callosum. The mean MD value of the splenium of corpus callosum was 0.709, 0.773, 0.823, and the standard deviation was 0.059. The number of patients to be enrolled in each group was calculated to be 8. Considering the test efficiency and the impact of shedding, 35 VCI patients, 35 N-VCI patients and 36 healthy individuals were finally included.

### Statistical Analysis

Statistical analyses were performed using SPSS 11.0. Data are presented as mean ± standard deviation (SD). One-way analysis of variance was used to assess quantitative differences across the three study groups. The least squares difference method was used to compare factors that satisfied the homogeneity of variance criteria among groups. The Dunnett T3 test was used to compare data that did not meet the homogeneity of variance criteria among groups. Correlations between MMSE score and the DKI diffusivity and kurtosis parameters were analyzed using Pearson correlation analysis. Receiver operating characteristic (ROC) curves were used to analyze significantly associated parameters; area under the curve (AUC), specificity, and sensitivity were calculated separately. ROC curves were analyzed to assess the discriminatory power of measured parameters to predict VCI. All reported *P*-values are two-tailed. *P* < 0.05 was considered statistically significant.

## Results

### General Characteristics, Cognitive Function, and Neuropsychological Evaluation

There were no significant differences in age, sex, education level, history of hypertension, history of diabetes, history of smoking, alcohol consumption, or blood biochemical indices among the three groups. Further, NIHSS scores, MRS scores, and sizes and locations of cerebral infarction were comparable between the VCI and N-VCI groups, with no significant difference between the two groups (*P* > 0.05) (Table 1).

**Table 1:** General characteristics of the study subjects.

		**N-VCI group (*N* = 35)**	**VCI group (*N* = 35)**	**NORM group (*N* = 36)**	***X^**2**^/T/F***	***P***
Age (years)		66.34 ± 11.67	63.09 ± 9.59	61.11 ± 11.37	2.073	0.131
Gender	Male	17 (48.6%)	15 (42.9%)	21 (58.3%)	1.743	0.418
	Female	18 (51.4%)	20 (57.1%)	15 (41.7%)		
Duration of education	≤ 9 years	20 (57.1%)	16 (45.7%)	20 (55.6%)	1.080	0.583
	≥9 years	15 (42.9%)	19 (54.3%)	16 (44.4%)		
Hypertension	Have	30 (85.7%)	28 (80.0%)	24 (66.7%)	3.884	0.143
	No	5 (14.3%)	7 (20.0%)	12 (33.3%)		
Diabetes Mellitus	Have	17 (48.6%)	21 (60.0%)	17 (47.2%)	1.391	0.499
	No	18 (51.4%)	14 (40.0%)	19 (52.8%)		
History of smoking	Have	16 (45.7%)	16 (45.7%)	19 (52.8%)	0.475	0.789
	No	19 (54.3%)	19 (54.3%)	17 (47.2%)		
History of drinking	Have	12 (34.3%)	15 (42.9%)	18 (50.0%)	1.797	0.407
	No	23 (65.7%)	20 (57.1%)	18 (50.0%)		
NIHSS		2.14 ± 1.67	2.17 ± 2.55	–	−0.056	0.956
MRS		1.86 ± 1.38	1.51 ± 1.20	–	1.112	0.270
Size of the infarction	Lacunar infarction	20 (57.1%)	23 (65.7%)	–	3.209	0.201
	Small infarction	15 (42.9%)	10 (28.6%)	–		
	Large infarction	0 (0%)	2 (5.7%)	–		
Lobes of the infarction	Frontal lobes	7 (20%)	11 (31.4%)	–	2.303	0.512
	Temporal lobes	7 (20%)	9 (25.7%)	–		
	Parietal lobes	7 (20%)	6 (17.1%)	–		
	Occipital lbes	14 (40%)	9 (25.7%)	–		
Locations of the infarction	Corpus callosum	6 (17.1%)	8 (22.9%)	–	2.209	0.530
	Corona radiata	11 (31.4%)	7 (20%)	–		
	Thalamus	15 (42.9%)	14 (40%)	–		
	Subcortic-al white matter	3 (8.6%)	6(17.1%)	–		
	Cerebral cortex	0 (0%)	0 (0%)	–		
Glucose (mmol/L)		7.35 ± 4.03	7.68 ± 4.22	6.05 ± 2.75	1.911	0.153
Glycated hemoglobin (%)		7.33 ± 2.29	7.24 ± 2.16	6.99 ± 2.06	0.236	0.791
UA (umol/L)		257.95 ± 68.95	264.37 ± 67.31	277.66 ± 74.87	0.724	0.487
Cr (umol/L)		68.56 ± 11.79	67.10 ± 13.98	66.18 ± 10.94	0.338	0.714
TC (mmol/L)		4.06 ± 0.91	4.57 ± 1.19	4.18 ± 0.85	2.487	0.088
TG (mmol/L)		1.35 ± 1.57	1.74 ± 2.11	1.59 ± 1.52	0.447	0.641
LDL-C (mmol/L)		2.33 ± 0.70	2.47 ± 0.80	2.23 ± 0.60	1.028	0.361
HDL-C (mmol/L)		1.12 ± 0.24	1.19 ± 0.24	1.25 ± 0.29	2.069	0.131
Hs-CRP (mg/L)		0.88 ± 0.77	0.90 ± 0.42	0.85 ± 0.53	0.057	0.944
HCY (umol/L)		12.05 ± 2.74	12.49 ± 2.98	11.77 ± 2.58	0.605	0.548
TSH (mIU/L)		2.45 ± 1.02	2.26 ± 1.02	2.29 ± 0.98	0.372	0.690
FT3 (pmol/L)		4.61 ± 0.64	4.56 ± 0.57	4.66 ± 0.58	0.234	0.792
FT4 (pmol/L)		14.39 ± 0.83	14.10 ± 1.20	14.28 ± 1.85	0.404	0.609
Vitamin B12		326.76 ± 46.89	325.33 ± 64.34	324.77 ± 65.77	0.010	0.990
Folic acid		5.73 ± 2.13	6.60 ± 2.85	6.68 ± 2.10	1.727	0.183

*NIHSS, National Institutes of Health Stroke Scale; MRS, Modified Ranking Scale; VCI, vascular cognitive impairment; N-VCI, non-vascular cognitive impairment; UA, Uric Acid; Cr, Creatinine; TG, Triglyceride; TC, Total cholesterol; LDL-C, Low density lipoprotein; HDL-C, High-density lipoprotein; Hs-CRP, Hypersensitive C-reactive protein; HCY, Homocysteine; TSH, Thyroid Stimulating Hormone; FT3, Free triiodothyronine; FT4, Free thyroxine; P-value is the comparison result among the three groups*.

As shown in Table 2, there were significant differences in MMSE score among the three groups, with those of the VCI group lowest and those of the NORM group highest. MMSE scores were divided into five parts: orientation, registration, attention and calculation, recall, and language and praxis. The VCI group scored worse than the N-VCI group and the NORM group in all five MMSE components, while there was no significant difference in HAMA and HAMD scores among the three groups.

**Table 2:** Cognitive function and neuropsychological evaluation of the study subjects.

		**N-VCI group (*N* = 35)**	**VCI group (*N* = 35)**	**NORM group (*N* = 36)**	***F***	***P***
MMSE	Total score	28.43 ± 0.739^*^	21.63 ± 2.860^*^#	29.11 ± 0.747	196.270	<0.001
	Orientation	9.83 ± 0.382	8.89 ± 1.132^*^#	10.00 ± 0.000	26.944	<0.001
	Registration	2.89 ± 0.323	1.94 ± 0.482^*^#	2.89 ± 0.319	71.874	<0.001
	Attention and Calculation	4.86 ± 0.355	3.60 ± 0.553^*^#	5.00 ± 0.000	146.602	<0.001
	Recall	2.06 ± 0.236	0.91 ± 0.445^*^#	2.22 ± 0.637	80.578	<0.001
	Language and Praxis	8.80 ± 0.473	6.29 ± 1.152^*^#	9.00 ± 0.000	157.310	<0.001
HAMA		4.09 ± 2.161	4.40 ± 1.943	3.67 ± 2.230	1.074	0.345
HAMD		4.86 ± 2.060	5.20 ± 1.891	5.89 ± 2.315	2.232	0.112

MMSE, Mini-Mental State Examination; HAMA, Hamilton Anxiey Scale; HAMD, Hamilton Depression Scale; P-value is a comparison among the three groups; Significantly different (^*^p < 0.05 vs. NORM group), (#p < 0.05 vs. N-VCI group).

### Comparisons of ROI DKI Parameters Among the Three Groups

**Figure 2:**
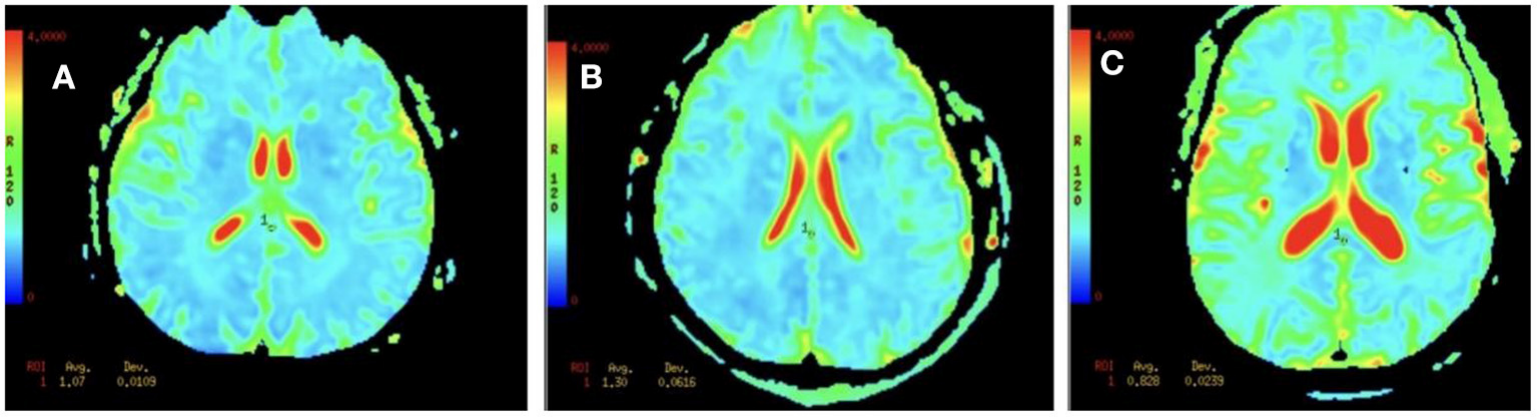
MD values for the splenium of corpus callosum in an N-VCI patient **(A)**, a VCI patient **(B)**, and a healthy control **(C)**. Mean diffusion (MD) maps was generated for each subject. These maps were aligned and ROI was manually drawn for the splenium of corpus callosum (Table 1), and the metric values were recorded. Our results showed that the MD values in the splenium of corpus callosum in the VCI group were higher than those in N-VCI and NORM groups (*P* < 0.05).

**Figure 3:**
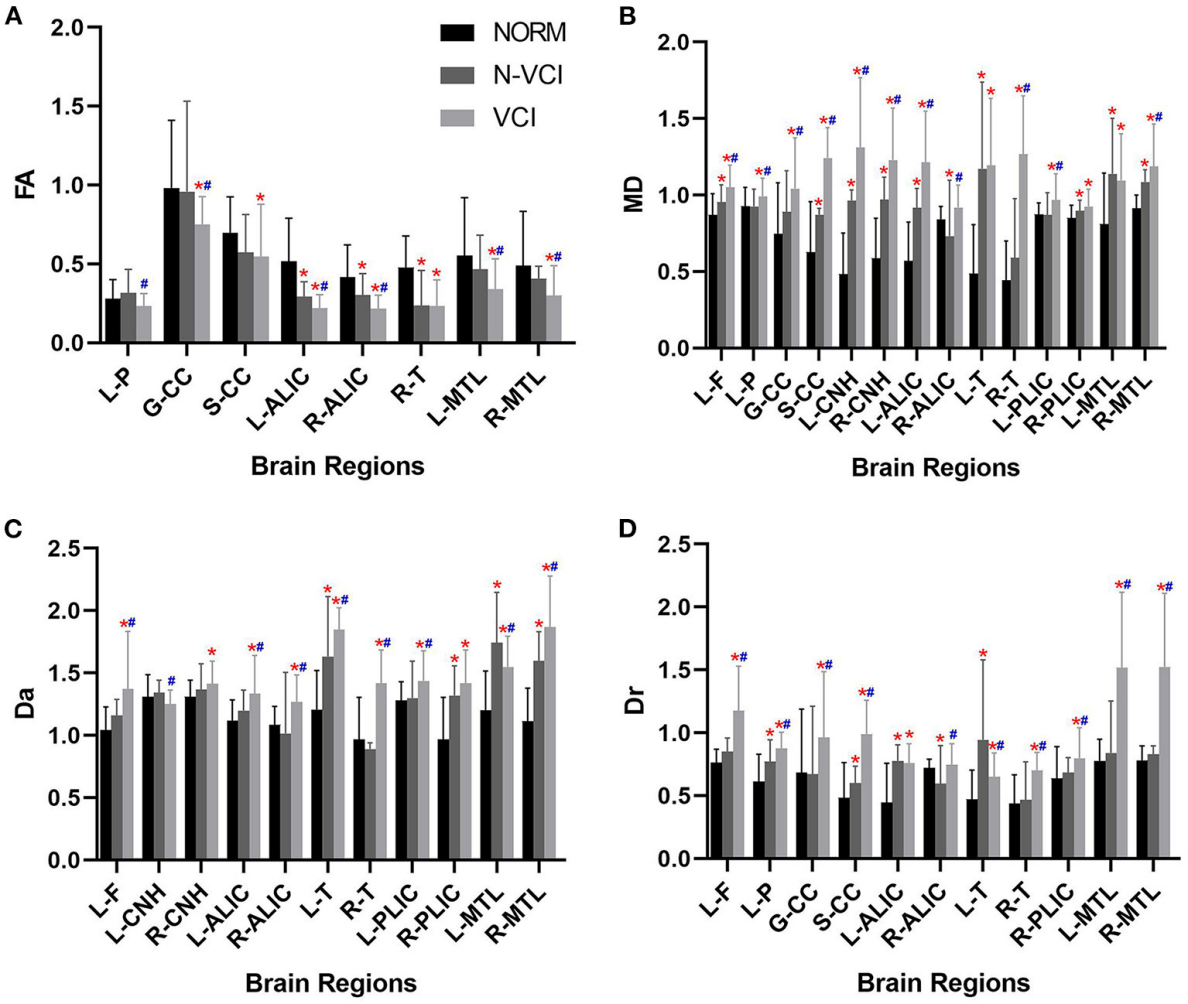
Means (± SD) of diffusivity metrics in each ROI, for each study group. **(A)** FA, **(B)** MD, **(C)** Da, and **(D)** Dr indices are indicated with significant differences (Tukey's multiple comparison correction). VCI patients had significantly higher diffusivity metrics in most ROIs than the N-VCI and NORM groups. FA, fractional anisotropy; MD, mean diffusion; Da, axial diffusion; Dr, radial diffusion. ROIs: L-F, left frontal lobe; L-P, left parietal lobe; GCC, genu of corpus callosum; SCC, splenium of corpus callosum; L-CNH, left head of caudate nucleus; R-CNH, right head of caudate nucleus; L-ALIC, anterior limb of left internal capsule; R-ALIC, anterior limb of right internal capsule; L-T, left thalamus; R-T, right thalamus; L-PLIC, posterior limb of left internal capsule; R-PLIC, posterior limb of right internal capsule; L-MTL, left medial temporal lobe; R-MTL, right medial temporal lobe. Significant differences are indicated as follows: *p < 0.05 vs. NORM group and #*p* < 0.05 vs. N-VCI group. N-VCI, non-vascular cognitive impairment; VCI, vascular cognitive impairment; NORM, healthy control.

**Figure 4:**
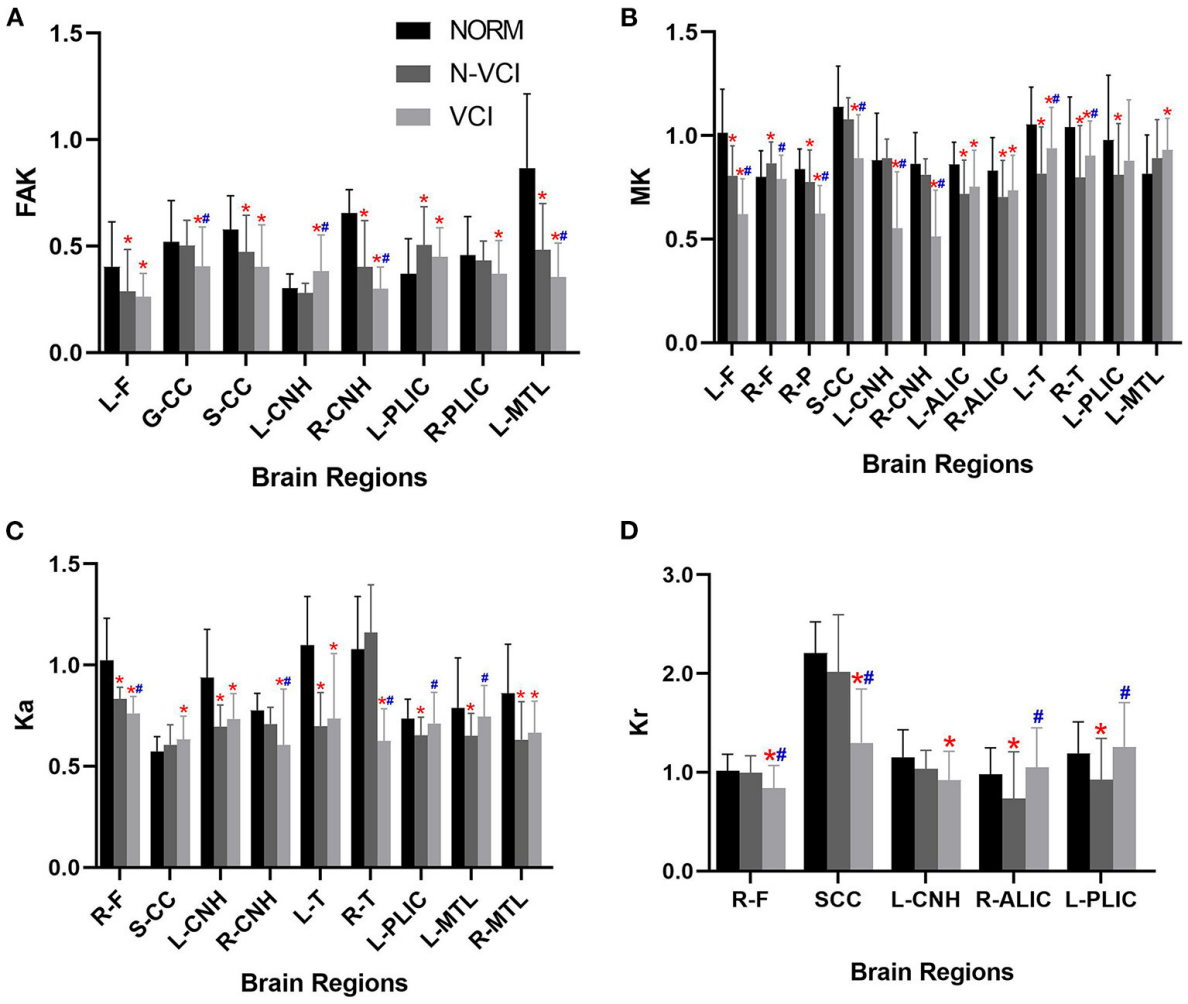
Means (± SD) of kurtosis metrics in each ROI, for each study group. **(A)** FAK, **(B)** MK, **(C)** Ka, and **(D)** Kr indices are indicated with significant differences (Tukey's multiple comparison correction).VCI patients had significantly lower kurtosis metrics in most ROIs than the N-VCI and NORM groups. FAK, fractional anisotropy kurtosis; MK, mean kurtosis; Ka, axial kurtosis; Kr, radial kurtosis. ROIs, L-F, left frontal lobe; R-F, right frontal lobe; R-P, right parietal lobe; GCC, genu of corpus callosum; SCC, splenium of corpus callosum; L-CNH, left head of caudate nucleus; R-CNH, right head of caudate nucleus; L-ALIC, anterior limb of left internal capsule; R-ALIC, anterior limb of right internal capsule; L-T, left thalamus; R-T, right thalamus; L-PLIC, posterior limb of left internal capsule; R-PLIC, posterior limb of right internal capsule; L-MTL, left medial temporal lobe; R-MTL, right medial temporal lobe. Significant differences are indicated as follows: *p < 0.05 vs. NORM group and #*p* < 0.05 vs. N-VCI group. N-VCI, non-vascular cognitive impairment; VCI, vascular cognitive impairment; NORM, healthy controls.

The mean MD value for the splenium of corpus callosum for the VCI group was significantly higher than those of the N-VCI and NORM groups (*P* < 0.05) (Figures 2, 3). Further, VCI patients had significantly higher diffusivity metrics and significantly lower kurtosis metrics in most ROIs than the N-VCI and NORM groups; however, some ROIs did not conform to this pattern (detailed size relationships are summarized in Figures 3, 4).

No parameters of the bilateral temporal lobe, bilateral occipital lobe, bilateral putamen, bilateral corona radiata, bilateral subcortical white matter, or bilateral cerebral cortex differed significantly among the three groups.

### Correlations Between DKI Parameters and MMSE Score

DKI values in all ROIs, other than the bilateral temporal lobe, bilateral occipital lobe, bilateral putamen, bilateral corona radiata, bilateral subcortical white matter, and bilateral cerebral cortex, were significantly correlated with MMSE score (Table 3). There were significant positive correlations of MD, Da, and Dr with MMSE score and significant negative correlations of FA, FAK, MK, Ka, and Kr with MMSE score in most ROIs; however, there were exceptions in some ROIs, such as the left head of the caudate nucleus, left medial temporal lobe, and left thalamus (detailed Pearson's coefficient values are presented in Table 3).

**Table 3:** Pearson's correlations of DKI parameters with MMSE scores.

**Brain region**	**FA**	**MD**	**Da**	**Dr**	**FAK**	**MK**	**Ka**	**Kr**
L-F	−0.008	−0.350^**^	−0.365^**^	−0.498^**^	0.276^**^	0.523^**^	−0.119	−0.084
R-F	0.187	0.044	0.082	0.057	0.028	0.105	0.411^**^	0.301^**^
L-P	0.176	−0.113	0.074	−0.330^**^	−0.059	0.007	−0.068	−0.037
R-P	0.181	−0.159	−0.006	−0.222^**^	−0.002	0.466^**^	0.009	−0.021
G-CC	0.257^**^	−0.324^**^	−0.019	−0.124	0.266^**^	−0.037	0.064	−0.076
S-CC	0.220^**^	−0.652^**^	0.090	−0.629^**^	0.257^**^	0.405^**^	−0.028	0.550^**^
L-CNH	0.179	−0.587^**^	0.261^**^	0.027	−0.324^**^	0.559^**^	0.192^**^	0.341^**^
R-CNH	0.003	−0.597^**^	−0.164	−0.145	0.509^**^	0.608^**^	0.285^**^	0.010
L-ALIC	0.441^**^	−0.579^**^	−0.269^**^	−0.304^**^	−0.073	0.192^**^	−0.106	−0.030
R-ALIC	0.383^**^	−0.169	−0.188	−0.140	0.064	0.046	−0.146	−0.084
L-T	0.045	−0.348^**^	−0.418^**^	0.030	−0.175	−0.008	0.258^**^	−0.252^**^
R-T	0.234^**^	−0.672^**^	−0.504^**^	−0.384^**^	−0.119	0.054	0.612^**^	0.177
L-PLIC	0.008	−0.268^**^	−0.165	−0.206^**^	−0.094	0.053	−0.030	−0.123
R-PLIC	0.160	−0.137	−0.295^**^	−0.264^**^	0.232^**^	0.198^**^	−0.040	−0.098
L-MTL	0.248^**^	−0.110	−0.097	−0.559^**^	0.402^**^	−0.201^**^	0.018	−0.076
R-MTL	0.2744^**^	−0.465^**^	−0.567^**^	−0.622^**^	0.040	−0.149	0.082	−0.039

*L-F, left frontal lobe; R-F, right frontal lobe; L-P, left parietal lobe; R-P, right parietal lobe; L-ALIC, the anterior limb of left internal capsule; R-ALIC, the anterior limb of right internal capsule; L-PLIC, the posterior limb of left internal capsule; R-PLIC, the posterior limb of right internal capsule; G-CC, the genu of corpus callosum; S-CC, the splenium of corpus callosum; L-CNH, left head of caudate nucleus; R-CNH, right head of caudate nucleus; L-T, left thalamus; R-T, right thalamus; L-MTL z, left medial temporal lobe; R-MTL, right medial temporal lobe*.

* *P < 0.05*,

** *P < 0.01*.

### ROC Curve Analysis

**Figure 5:**
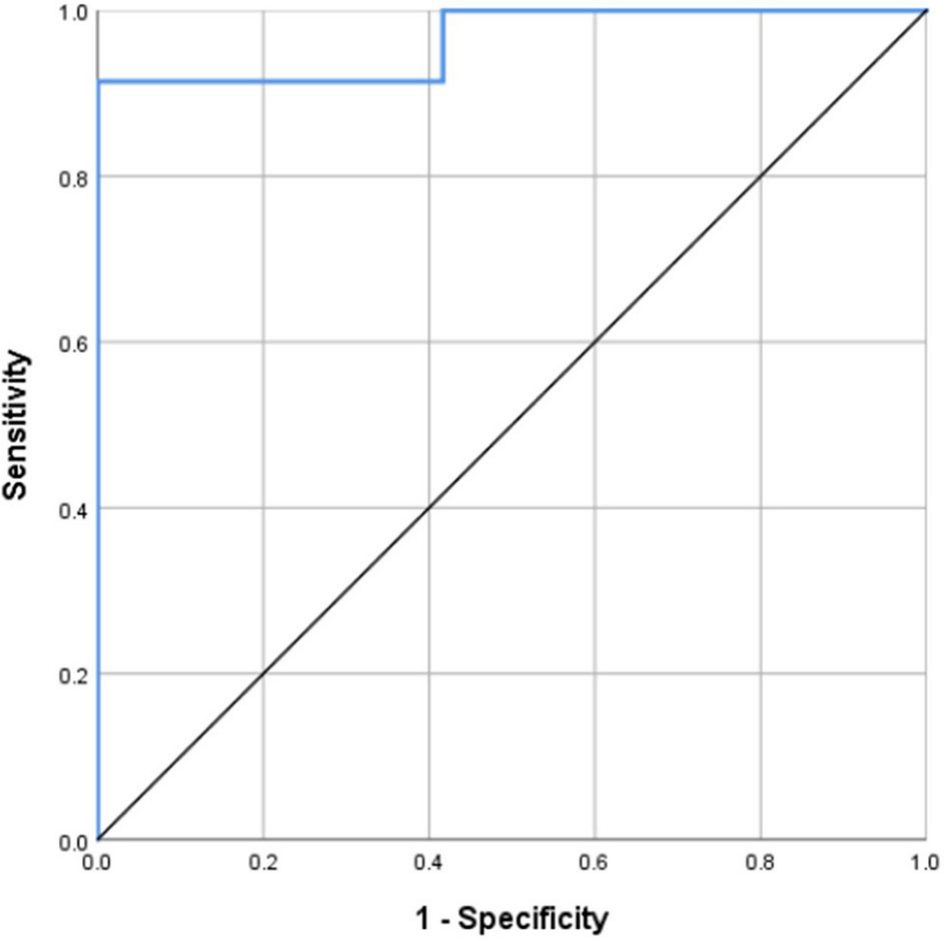
Receiver operating characteristic (ROC) curves for MD of the splenium of corpus callosum, distinguishing VCI from NORM. MD in the splenium of the corpus callosum was identified as a good individual discriminator of VCI from healthy controls using ROC analysis; at the optimal cut-off MD value of 0.9915, sensitivity and specificity were 91.4 and 100%, respectively, with an AUC value of 0.964.

MD in the splenium of the corpus callosum was identified as a good individual discriminator of VCI from healthy controls using ROC analysis; at the optimal cut-off MD value of 0.9915, sensitivity and specificity were 91.4 and 100%, respectively, with an AUC value of 0.964 (Figure 5).

### Correlations Between Diffusivity Parameters and Kurtosis Parameters

**Figure 6:**
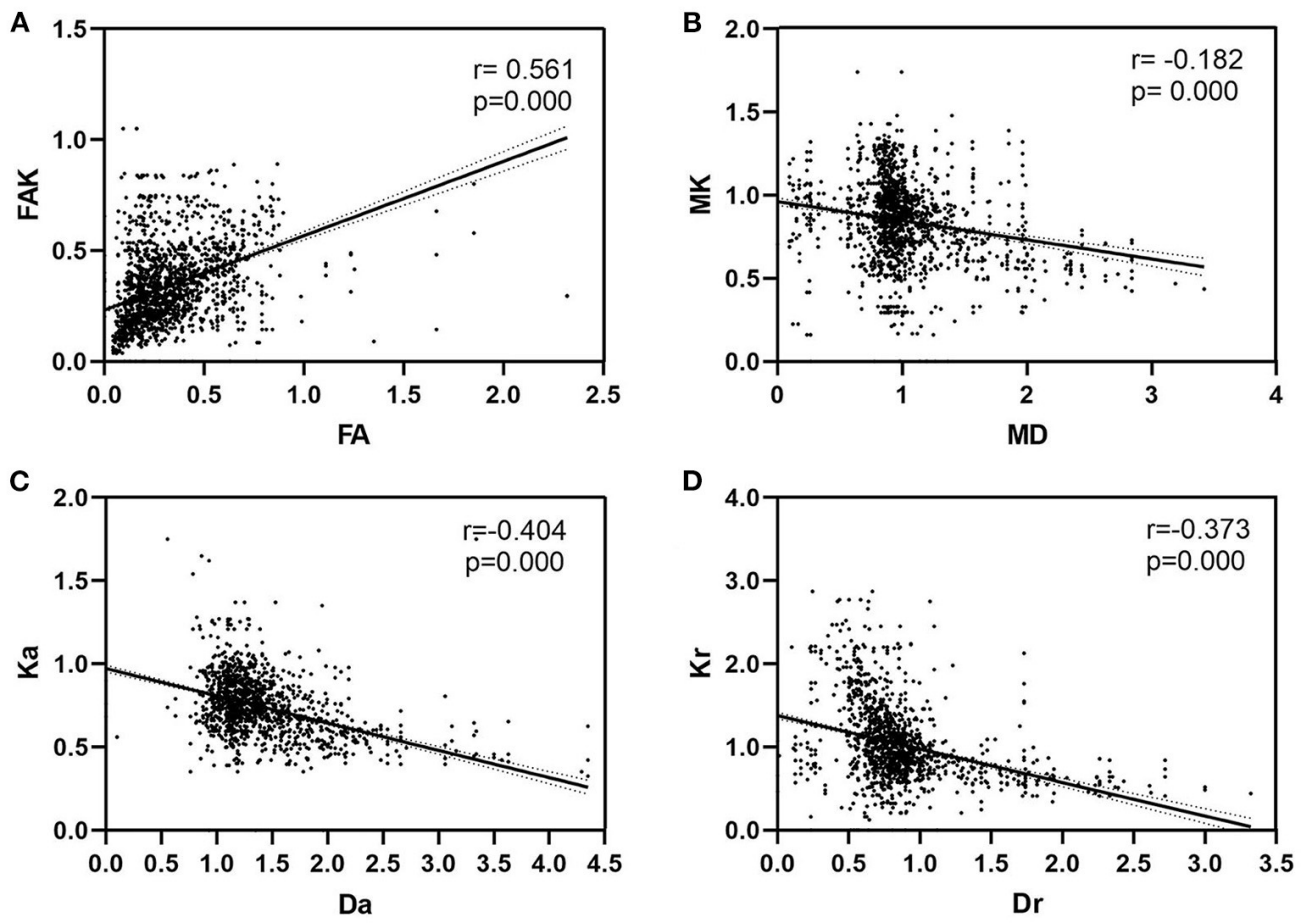
Correlations between diffusivity parameters and kurtosis parameters. **(A)** There were positive correlations between FA and FAK in all ROIs (*P* < 0.01). Further, **(B)** there were significant negative correlations between MD and MK (*P* < 0.01), **(C)** Da and Ka (*P* < 0.01), and **(D)** Dr and Kr (*P* < 0.01). FA, fractional anisotropy; MD, mean diffusion; Da, axial diffusion; Dr, radial diffusion; FAK, fractional anisotropy kurtosis; MK, mean kurtosis; Ka, axial kurtosis; Kr, radial kurtosis.

There were positive correlations between FA and FAK in all ROIs (*r* = 0.561, *P* < 0.01). Further, there were significant negative correlations between MD and MK (*r* = −0.182, P < 0.01), Da and Ka (*r* = −0.404 and *P* < 0.01), and Dr and Kr (*r* = −0.373 and *P* < 0.01) (Table 4 and Figure 6).

**Table 4:** Pearson's correlations between diffusivity parameters and kurtosis parameters.

	**FA**	**MD**	**Da**	**Dr**
FAK	0.561^**^	–	–	–
MK	–	−0.182^**^	–	–
Ka	–	–	−0.404^**^	–
Kr	–	–	–	−0.373^**^

*FA, fractional anisotropy; MD, mean diffusion; Da, axial diffusion; Dr, radial diffusion; FAK, fractional anisotropy kurtosis; MK, mean kurtosis; Ka, axial kurtosis; Kr, radial kurtosis*.

** *P < 0.01*.

## Discussion

In this study, no significant differences in age, sex, years of education, or vascular risk factors were detected among the N-VCI, VCI, and NORM groups, and there were no significant differences in the size, location, or severity of cerebral infarction between VCI and N-VCI groups; hence, the effects of possible confounding factors on cognitive assessment were largely eliminated. In addition, there were no significant differences in DKI parameters among the three groups in the corona radiate, subcortical white matter, or cerebral cortex, which largely eliminated the influence of acute cerebral infarction on DKI parameters, increasing confidence in our findings. Therefore, in a situation where there were no significant differences in potential confounding factors among the three groups, we found that FA values in the genu of corpus callosum, the anterior limb of bilateral internal capsule, and bilateral medial temporal lobe were lower in the VCI group than the N-VCI and healthy control groups, while MD values in most ROIs were higher. In previous studies, Chen et al. (14), Zhou et al. (15), and Wei et al. (16) reported that mean FA from DTI was significantly lower and MD from DTI significantly higher in the VCI group than in N-VCI and healthy control groups. Our results are consistent with these previous studies, as we discovered that FA values from DKI were lower in some ROIs, and the MD values from DKI higher, in the VCI group than the N-VCI and healthy control groups. FA reflects the degree of arrangement and structural integrity of cell structures in a fiber bundle, while MD reflects the overall diffusion level and diffusion resistance of whole molecules (9, 15). Although the specific cellular mechanisms underlying differences in these two parameters in VCI are unclear (17, 18), based on our findings we speculate that the abnormal diffusion coefficient indices observed in patients with VCI can be explained by axon demyelination, loss of neurons, and increased water channel activity, leading to reduced water diffusion. This hypothesis is supported by earlier studies in which diffuse white matter changes, with myelin loss and axonal abnormalities, have been recorded in almost all types of vascular dementia (19).

Previous studies on VCI focused only on DTI, and highlighted diffusion indicators, such as FA, MD, DA, and Dr, while related studies (20) demonstrated that a combination of kurtosis and diffusion indices can better reveal brain tissue signal heterogeneity, facilitating detection of brain tissue microstructure changes. Therefore, compared with previous studies, we added kurtosis indices, including FAK, MK, Ka, and Kr, and further expanded the ROI, with the aim of revealing more comprehensive and concrete changes in brain microstructure in patients with VCI. We found that the MK values of some ROIs in the VCI group were lower than those in N-VCI and healthy control groups, while in bilateral thalamus, MK values were lower in VCI group than those in healthy control group, but higher than those in the N-VCI group. To date, there have been very few DKI studies on VCI, as most previous DKI studies have focused on mild cognitive impairment (MCI) and AD. Falangolael et al. (21), Wang et al. (22), and Liu et al. (23) found that, relative to healthy controls, kurtosis indices were lower in patients with MCI and AD. In our study, we discovered that MK values in some ROIs of patients with VCI were lower than those of the N-VCI and healthy control groups, while in others ROIs, MK values were higher in patients with VCI than those of the N-VCI group. While the exact meaning of and pathological basis for kurtosis have yet to be fully elucidated (24, 25). To date, the general reason for kurtosis has been considered microstructure complexity (26), where the more complex and intricate a structure is, the higher the degree of non-Gaussian water molecule diffusion, and the greater the MK value (27, 28). Some scholars have postulated that increases and decreases in kurtosis indicate potential disease changes or microstructure, and hence that kurtosis can provide rich microstructure information, with extensive analysis of pathological kurtosis potentially providing more information than diffusion coefficient data (29, 30). The advantages of DKI have been supported by studies for other clinical applications, where it detected more abnormal areas than DTI (31, 32). In this study, we hypothesized that kurtosis indices could provide more specific indications of VCI pathology. Neuroinflammation, neuronal swelling, and glial proliferation may all influence brain tissue microstructural complexity in VCI, in addition to demyelination and axonal degeneration. Therefore, we predicted that important changes in kurtosis would be able to distinguish early changes involved in VCI.

Previous studies had identified certain correlations between DKI parameters and cognitive function (30, 31); Bozzali et al. (33) and Morikawa et al. (34) reported a significant correlation between FA and MMSE score, while Yoshiura et al. (35) demonstrated a correlation of MMSE score with MD, rather than FA. Our findings demonstrate that FA, FAK, MK, Ka, and Kr are positively correlated with MMSE score in most ROIs, while MD, Da, and Dr were negatively correlated with MMSE score. The strong associations between MMSE score and DKI parameters suggest that patient cognitive impairment in VCI may be due to brain tissue axonal loss or demyelination, as well as other microstructural alterations; however, interestingly, our study revealed bilateral inconsistency in the microstructural changes of the frontal lobes and their correlations with MMSE score. Microstructural changes in the left frontal lobe are more obvious than those in the right in patients with VCI, which may reflect the frontal lobe asymmetry routinely detected in healthy people. A study of asymmetry of the human brain showed a larger total surface area in the right than the left cerebral hemisphere (36); therefore, we speculate that the left frontal lobe is more vulnerable to microstructural changes in patients with VCI.

In addition, we found that splenium of corpus callosum MD had the strongest correlation with MMSE score (correlation coefficient, −0.652), further confirming that MD has better diagnostic value for VCI than other DKI parameters. Moreover, we conducted ROC curve analysis, which showed that, when the threshold MD value of the splenium of corpus callosum was set at 0.9915, the diagnostic AUC value of VCI was 0.964, with sensitivity and specificity values of 91.4 and 100%, respectively. No previous report has identified splenium of corpus callosum MD as a potential diagnostic indicator of VCI. It is established that acute cerebral infarction can lead to ischemia and hypoxia of tissue across the whole brain, leading to dysfunctional myelinogenesis and nerve fiber damage. The corpus callosum is made up of fibers that originate from large pyramidal neurons and is one of the most extensively myelinated areas of the brain. Therefore, pathological changes often occur in the corpus callosum, even when ischemia or hypoxia is mild. Previous studies reported that the strongest associations between abnormal DTI parameters and cognitive decline were in the corpus callosum and corona radiata (14, 37–41). In our study, we also found that abnormalities of the corpus callosum and DKI parameters were strongly represented.

Of all DKI parameters in all ROIs, we observed that MD was significantly negatively correlated with MK, Da with Ka, Dr with Kr, and that FA was significantly positively correlated with FAK, which led us to hypothesize that changes in diffusivity were accompanied by alterations in diffusional non-Gaussianity. Liu et al. (23) found that, in addition to a correlation between Da and Ka, there were correlations between Dr and Kr, MD and MK, and FA and FAK in patients with cerebrovascular disease complicated with MCI. Gong et al. (42) found that MD and MK, Da and Ka, and Dr and Kr were significantly negatively correlated when observing changes in white matter and gray matter DKI parameters in patients with MCI and AD. The combination of kurtosis and diffusion in the DKI coefficient matrix provides two perspectives on microstructure by integrating non-Gaussian and Gaussian processes into one model. Hence, combination of these two features can represent the whole microstructure. Although variations in kurtosis parameters can be partly attributed to radial or axial variation of the absolute diffusion coefficient, they cannot be completely explained by these variations. Therefore, while kurtosis parameters represent the geometric distribution of diffusion, the neurobiological mechanisms leading to changes in kurtosis remain unclear.

This study has certain limitations. First, our preliminary findings were primarily used to determine whether DKI is useful as a new imaging tool for assessing changes in brain microstructure in patients with VCI; however, our sample size requires expansion and long-term longitudinal follow-up studies are needed to verify the long-term diagnostic significance of DKI parameters in patients with VCI. Second, because of the limited number of patients with VCI included in this study, we did not conduct further analysis according to VCI classification; therefore, we need to expand the sample size to allow VCI grading and more accurate evaluation of the significance of DKI parameters in patients with VCI. Third, although interesting changes in kurtosis parameters were observed in the frontal lobe, parietal lobe, internal capsule, corpus callosum, head of caudate nucleus, thalamus, and medial temporal lobe, the potential pathophysiological significance of these parameters requires further study. Finally, previous studies (43, 44) had combined electroencephalogram (EEG) with DTI, and provided a methodology to access specific networks of subcortical fiber tracts subserving the maintenance of interhemispheric resting state coherence in the human brain. Therefore, we can further combine DKI with EEG, that may be a more accurate method to evaluate patients with VCI.

In conclusion, pathological changes in some brain regions may underlie cognitive impairment after acute cerebral infarction, especially the splenium of corpus callosum. These preliminary results suggest that, in patients with VCI, DKI may be useful for assessing microstructural tissue damage. For early diagnosis of changes in white matter microstructure in patients with VCI, DKI provides a comprehensive imaging reference, and DKI-derived parameters may be a feasible means of evaluating patients with VCI. In addition, some DKI parameters are significantly correlated with patient clinical evaluation scores. Therefore, DKI-derived parameters may be a viable method to evaluate patients with VCI, and to predict potential brain structure imaging biomarkers of VCI progress.

## Data Availability Statement

The raw data supporting the conclusions of this article will be made available by the authors, without undue reservation.

## Ethics Statement

The studies involving human participants were reviewed and approved by the Second Hospital of Dalian Medical University. The patients/participants provided their written informed consent to participate in this study.

## Author Contributions

LF, FEI, and YF contributed to the preparation of the manuscript. LF, XC, HY, contributed to the data collection. LF, ZW, CT, XC, YM, and YG contributed to data analysis and interpretation. LF, YD, CY, and YG contributed to the experimental design and manuscript revision. All authors contributed substantially to this work and approved the final manuscript.

## Funding

This study was supported by the National Natural Science Foundation of China 81800823, Natural Science Foundation of Liaoning Province 20180530015, United Fund of the Second Hospital of Dalian Medical University and Dalian Institute of Chemical Physics, Chinese Academy of Sciences (UF-ZD-202012).

## Conflict of Interest

The authors declare that the research was conducted in the absence of any commercial or financial relationships that could be construed as a potential conflict of interest.

## Publisher's Note

All claims expressed in this article are solely those of the authors and do not necessarily represent those of their affiliated organizations, or those of the publisher, the editors and the reviewers. Any product that may be evaluated in this article, or claim that may be made by its manufacturer, is not guaranteed or endorsed by the publisher.
